# Soluble HER-2 in Patients with Head and Neck Squamous Cell Carcinoma

**Published:** 2013-06

**Authors:** Bijan Khademi, Behzad Khademi, Abbas Ghaderi, Seyd Fakhroddin Hosseini, Nika Niknejad

**Affiliations:** 1 *Department of Otorhinolaryngology, Shiraz Institute for Cancer Research, Shiraz University of Medical Sciences, Shira, Iran.*; 2 *Department of ophthalmology,Shiraz University of Medical Sciences.*; 3 *Department of Immunology, Shiraz Institute for Cancer Research, Shiraz University of Medical Sciences, Shira, Iran.*

**Keywords:** EGFR, HER-2, HNSCC, Tmor marker

## Abstract

**Introduction:**

The presence of HER-2 has been shown to be a prognostic factor in many kinds of cancers, but its role in head and neck squamous cell carcinoma (HNSCC) is not still defined. The purpose of the current study is to investigate the role of HER-2 in HNSCC and its correlation with various clinicopathologic parameters.

**Materials and Methods:**

Peripheral blood samples were obtained from 17 healthy volunteers and 69 patients with HNSCC before curative surgery. The HER-2 level was determined in each sample sandwich by ELISA. Statistical analysis was performed using an independent t-test, one-way ANOVA, and the Duncan procedure.

**Results:**

Mean HER-2 serum levels were higher in patients with HNSCC compared with healthy controls, although the difference was not statistically significant (3.85ng/ml vs. 3.75ng/ml; P>0.05). The mean serum level of HER-2 in patients with was higher in patients with lymph node involvement, metastasis, invasion, tumor size ≥2 cm, and stage>1, although the differences were not statistically significant (P>0.05).

**Discussion:**

Mean HER-2 serum levels in patients with tumor size T3 and higher were higher than those from patients in stage T1 and T2.over expression of these receptor translate into disease progression, growth and invasiveness, with the increase serum HER-2 levels in such patients offering some support for this theory.

**Conclusion:**

In this study the mean HER-2 serum level in patients with HNSCC was found to be greater in comparison with the healthy control group, although the difference was statistically insignificant. From the analysis of the results of the current study we have come to the conclusion that by increasing sample size the rising of the serum HER-2 level in patients with HNSCC can be meaningful. Apart from this, the role of HER-2 as a tumor marker in patients with HNSCC is still controversial and needs further studies to clarify the significance of this biomarker for early detection or screening for HNSCC.

## Introduction

Head and neck squamous cell carcinoma (HNSCC) is a debilitating and lethal malignancy with progressive and local spread affecting highly critical functions of speech, swallowing, and respiration. Overall this disease affects more than 500,000 people around the world (1,2). Despite aggressive multidisciplinary advances in surgery, chemotherapy, and radiotherapy the survival rate has only improved moderately, with the 5-year survival rate remaining at 50% over the past 30 years (3,4).

Patients with premalignant lesions and early stage cancers have a high rate of survival, but the vast majority of Stages III and IV cases are fatal, partly due to the relatively high local and regional recurrence rates. The biological factors that underlie the locoregional and distant spread of this neoplasm are not completely understood (5,6). Early detection of HNSCC could improve clinical outcomes, but there is no definite evidence that widespread population screening using conventional methods such as head and neck examination and fiber optic endoscopy with direct visualization decreases mortality from HNSCC (7). 

To improve patient outcomes, novel therapeutic strategies that are more effective in improving survival are urgently needed. It is known that HNSCC results from the multistep accumulation of heterogeneous and genetic changes in squamous cells. These changes progressively increase the ability of transferred cells to proliferate and invade (8). The heterogeneity of these changes explain why tumors at the same clinical stages and localization often show significant differences in their clinical outcomes and treatment responses (9-11). 

The development of reliable biomarkers and more effective therapeutic agents is necessary to improve patient outcomes. The use of biological markers in body fluids for molecular detection of cancer has been the subject of an increasing number of studies with the intent to improve overall screening accuracy and cost-effectiveness. Body fluids can potentially carry whole cells as well as protein, DNA, and RNA species that allow for the detection of cellular alterations in cancerous cells.

The major goals of any robust molecular detection and diagnostic strategy are to identify early tumors and to use the available biomarkers to prognosticate and risk stratify patients and predict therapeutic response to conventional treatments and therapeutic failures.

Tumor suppressor genes, oncogenes, cell proliferation markers, angiogenic markers, and cell adhesion molecules have all been studied as potential tools to predict the prognosis of patients with HNSCC (12). Epidermal growth factor receptor (EGFR) is a widely studied oncogene in HNSCC. This tyrosine kinase receptor belongs to the Erb B family of cell-surface receptors and has many downstream signaling targets associated with tumorgenesis. Once activated, the receptor can signal via multiple pathways which are related to cellular proliferation, apoptosis, invasion, angiogenesis, and metastasis (13,14).

Dysregulaton in the signaling of EGFR and its downstream targets commonly occurs in epithelial cancers, but also in over 80% of HNSCC cases (14,15). 

EGFR is a promising marker and prognosticator of disease, and understanding of its molecular biology has led directly to significant targeted therapies (15).

EGFR is a biomarker belonging to a cell-cycle accelerator and proliferation group, and is a transmembrane cell surface receptor that binds to some ligands activating the protein-kinase system, which regulates the signaling involved in cell proliferation and differentiation (16). It belongs to a family of four similar receptors HER-1(Erb-1), HER-2 (neu/Erb-B2), HER-3(Erb-B3) and HER-4 (Erb-B4), all of which all share a common structure, with an extracellular ligand-binding domain, a transmembrane domain. An intracytoplasmic tyrosine kinase domain (17-19) ligand binding to these receptors induces the formation of receptor homodimers and heterodimers, and thereby activates numerous downstream pathways regulating diverse processes including differentiation, migration, proliferation,and survival.

EGFR activation can enhance the malignant potential of the epithelial tissues (17). In some studies, EGFR over-expression has been correlated with poor prognosis in HNSCC patients (20), although these results have not been confirmed in other investigations (21). We therefore decided to investigate the role of HER-2 in HNSCC and its correlations to various clinicopathologic parameters. 

## Materials and Methods

Patients with a histopathologic diagnosis of primary HNSCC who were referred to our center over a period of 1 year were enrolled in this study. The control group consisted of healthy volunteers matched according to gender and age to the patient group. Patients and controls who showed signs of significant morbidity or acute medical problems such as chronic heart failure, active infection, presence of human immunodeficiency virus, hepatitis, or any immunosuppressive disease were excluded from the study. The protocol of this study was approved by the Ethics Committee of Shiraz University of Medical Sciences.

Clinical data such as age, gender, symptoms, location of tumor, TNM staging, and tobacco and alcohol habits were obtained through use of a questionnaire. Histological grading of the malignancy was based on two parameters from a recognized grading system; the degree of keratinization and nuclear pleomorphism. 

A 3-cm^3^ clot blood sample was taken from all patients enrolled in the study and centrifuged to obtain serum, which was then kept at −80 °C until all samples were ready to be analyzed. 

HER-2 serum levels were measured using the sandwich ELISA test in accordance to the manufacturer-recommended procedures. HER-2 ELISA kit was purchased from Bendermed, Austria.

Statistical analysis was carried out using SPSS software for Windows (16.0) package (SPSS, Chicago, IL). Mann-Whitney Test and one-way ANOVA test and t-Test were used for statistical calculations.

## Results

Patient information and clinicopathologic information are shown in Table 1. The sample consisted of 69 patients with primary HNSCC in varied head and neck locations and 17 healthy volunteers as the control group. 

The T-staging and the N-staging of the tumors were described according to the American Joint Committee on Cancer (AJCC) classification for HNSCC.

The mean age was 59 years (±1.6 years) and 54 years (±7.7years) in the patient and control groups respectively. Out of the 69 patients, seven (10.1%) were female and 62 (89.9%) were male. In the control group, two patients (11.7%) were female and 15 (88.3%) were male.

Serum levels of HER-2 in the two groups were compared, along with a multivariate analysis of HER-2 serum levels in the patient group according with lymph nodal involvement, tumor size, local invasion, tumor staging, gender, and tobacco use (Table. 1).

The mean HER-2 serum level was 3.85 (±3.75) ng/ml in the patient group and 3.75 (±3.25) ng/ml) in the control group (P>0.05). 

Of the 69 patients with HNSCC, 41 (59.4%) showed invasion to the surrounding tissues, with HER-2 serum levels of 3.97 (±5.0) ng/ml. The remaining 28 HNSCC patients (40.6%) with no adjacent tissue invasion had a mean HER-2 serum level of 3.18 (±1.63) ng/ml. Despite the increased mean HER-2 serum levels in the more severe cases, the difference was not statistically significant (P>0.05).

**Table 1:** Mean Serum Levels of HER-2 on Basis of Different Parameters in HNSCC

	Number	Percentage %	Mean serum HER-2 ng/ml	P-value
HNSCC Patients Healthy control	69 17	80.2% 19.8%	3.85 3.75	>0.05
Female patients Male patients	7 62	10.1% 89.9%	3.08 3.95	>0.05
Patients with l.n˟ involvementPatients without l.n. involvement	26 43	37.6% 62.4%	4.87 3.08	>0.05
Smokers Non-smokers	48 21	69.5% 30.5%	3.51 4.73	>0.05
Tumor with invasion Tumor without invasion	41 28	59.4% 40.6%	3.97 3.18	>0.05
Tumor>2cm 2cm<tumor<4cm Tumor>4cm	17 30 22	24.6% 43.4% 31.8%	3.33 2.97 5.23	>0.05
Stage 1 Stage 11 Stage 111 Stage 1V	8 13 30 18	12.5% 18.5% 43.4% 26.1%	2.68 3.68 3.28 4.73	>0.05
Tumor with distant metastasis Tumor without metastasis	16 53	23.1% 76.9%	6.83 3.17	>0.05

In the patient group, 26 patients (37.6%) had histologically proven lymph node involvement and 43 (62.4%) had no lymph node involvement. Mean HER-2 serum levels in patients with lymph node invasion was 4.87 ng/ml (SD, 6.54 ng/ml) compared with 3.08 ng/ml (SD,1.7 ng/ml) among patients with no lymph node involvement (P>0.05).

In the patient group, 17 patients (24.6%) presented with a tumor in which the largest dimension was≤2 cm, equivalent to T1 staging. Thirty (43.4%) cases had a tumor size between 2 and 4 cm (T2 staging). A tumor size >4 cm was seen in 22 patients (31.8%). 

Mean serum HER-2 levels in T1, T2, and T3 stages were 3.33 (±1.8) ng/ml, 1.8 (±1.8) ng/ml, and 2.97 (± 6.75) ng/ml, respectively (P>0.05).

In the patient group, 48 patients (69.5%) were smokers, while 21 (30.5%) were non-smokers. Mean HER-2 serum levels were 2.51 (±1.7) ng/ml and 4.73 (± 6.44) ng/ml, respectively (P>0.05).

Eight of the 69 cases (12.5%) in the patient group had stage 1 TNM disease, 13 (18.8%) had stage II, 30 (42.4%) had stage III, and 18 (26.1%) had stage IV disease, with HER-2 serum levels of 2.68 (±0.85) ng/ml, 3.68 (±2.14) ng/ml, 3.28 (± 1.63) ng/ml, and 4.73 (±6.13) ng/ml, respectively (P>0.05).

Of the 69 patients, 16 (23.1%) had distant metastasis at the time of referral, with a mean HER-2 serum level of 6.83 (± 9.49) ng/ml. The remaining 50 patients (76.9%) with no distant metastasis had a mean HER-2 serum level of 3.17 (±1.7) ng/ml (P>0.05).

## Discussion

In this study, serum HER-2 levels in HNSCC patients were subjected to a multivariate analysis regarding lymph node involvement, adjacent tissue tumor involve- ment, distant metastasis, tumor size, gender, and smoking habits.

Even though the mean HER-2 serum levels were numerically higher in patients with HNSCC compared with the control group, the difference was not statistically significant (P>0.05). This lack of significance is most probably due to the small sample size.

Patients in the HNSCC sample group with lymph nodal involvement had numerically higher mean HER-2 serum levels relative to patients with no nodal disease (P>0.05). This finding is not surprising given that lymph node activity is independent of EGFR activity, and the lack of statistical significance may be a reflection of the independence of the HER-2 expression from the lymph node activity.

The non-significant difference in serum HER-2 levels between male and female patients has also been documented in previous studies (20).

Mean serum HER-2 levels were numeri- cally higher in patients with more invasive disease, although the lack of a statistically significant finding suggests the need for further studies.

Expression of EGFR family members is highly regulated outside of the bone marrow. Expression is generally low, with increased expression in tumors commonly characteri- zed as over-expression, as it reflects an increase above the baseline expression encountered in the majority of tissues. HER-2 gene amplification and over-expression has been reported in approximately 30% of breast cancers and in several other tumors, including ovarian, gastric, and colorectal cancers (21–24). In HNSCC, HER-2 over-expression has been described previously, although reports on its clinical relevance are less conclusive (20,25,26).

Mean HER-2 serum levels in patients with tumor sizes T3 and higher were greater than those from patients in stages T1 and T2. Although the difference was not statistically significant (P>0.05), this may be interpreted as a linear relationship between tumor size and HER-2 activity as EGFR is a known activator of cancerous cell growth. Mean HER-2 serum levels had a linear relationship with TNM staging, with the highest level of HER-2 serum levels detected in stage IV TNM patients. This may be for the same reason as the elevation of serum HER-2 levels in HNSCC patients with invasive disease; HNSCC progression as a reflection of over- expression of HER-2. 

Our most controversial finding was the elevation of mean HER-2 serum levels in HNSCC patients who were non-smokers compared with those who smoked. As tobacco is a known carcinogenic factor in HNSCC, this finding needs to be further evaluated to be analyzed correctly.

In the current study, the elevation of serum HER-2 levels in patients with more progressive disease as indicated by larger sized tumors, higher TNM stages, invasion of surrounding tissue, and distant metastasis may be a reflection of the significant role HER-2 plays as an epidermal growth factor in the growth of squamous cell carcinomas. The changes that cause the differentiation of HER-2 from a proto-oncogene to an oncogene are then seen as the over-expression of the epidermal growth factor receptor. Over-expression of this receptor translates into disease progression, growth and invasiveness, with the increase serum HER-2 levels in such patients offering some support for this assumption. Even the increase of HER-2 serum levels in patients with lymph node involvement can be seen as over-expression of EGFR HER-2, reflected as SCC progression. However, the relative increase in HER-2 serum levels in non-smokers with HNSCC in our study group in comparison with the non-smokers needs further investigation.

## Conclusion

The prognostic significance of HER-2 expression in HNSCC remains to be elucidated. Some investigators have shown that there was no significant correlation between HER-2 over-expression and clinicopathologic factors (27-29).

Our study provides evidence that EGFR dysregulation is associated with tumor progression from dysplasia to full-blown cancer and stimulates invasiveness and is indicative of poor prognosis. The findings of current study are consistent with those previous reports, (26,27,29), although conflicting outcomes have been also reported.

We conducted the current study to investigate whether these proteins can be considered as biomarkers that could guide therapeutic choices in HNSCC. On the basis of our findings it seems that the potential of HER-2 as a tumor marker in patients with HNSCC needs further studies. HER-2 can be heralded as a prognostic factor, as higher levels of this EGFR in serum seem to be associated with more severe disease; lymph node involvement, distant metastasis, increased tumor size and higher TNM stages of SCC. HER-2 over-expression can be indicative of an unfavorable prognosis. Further large-scale studies are needed to further document our findings, especially the prognostic value of HER-2 in HNSCC. 
